# The food and environmental safety of *Bt* crops

**DOI:** 10.3389/fpls.2015.00283

**Published:** 2015-04-29

**Authors:** Michael S. Koch, Jason M. Ward, Steven L. Levine, James A. Baum, John L. Vicini, Bruce G. Hammond

**Affiliations:** Monsanto Company, Saint LouisMO, USA

**Keywords:** *Bacillus thuringiensis*, Cry proteins, genetically modified crops, safety assessment, food and feed safety

## Abstract

*Bacillus thuringiensis* (*Bt*) microbial pesticides have a 50-year history of safety in agriculture. Cry proteins are among the active insecticidal ingredients in these pesticides, and genes coding for Cry proteins have been introduced into agricultural crops using modern biotechnology. The Cry gene sequences are often modified to enable effective expression in planta and several Cry proteins have been modified to increase biological activity against the target pest(s). Additionally, the domains of different but structurally conserved Cry proteins can be combined to produce chimeric proteins with enhanced insecticidal properties. Environmental studies are performed and include invertebrates, mammals, and avian species. Mammalian studies used to support the food and feed safety assessment are also used to support the wild mammal assessment. In addition to the NTO assessment, the environmental assessment includes a comparative assessment between the *Bt* crop and the appropriate conventional control that is genetically similar but lacks the introduced trait to address unintended effects. Specific phenotypic, agronomic, and ecological characteristics are measured in the *Bt* crop and the conventional control to evaluate whether the introduction of the insect resistance has resulted in any changes that might cause ecological harm in terms of altered weed characteristics, susceptibility to pests, or adverse environmental impact. Additionally, environmental interaction data are collected in field experiments for *Bt* crop to evaluate potential adverse effects. Further to the agronomic and phenotypic evaluation, potential movement of transgenes from a genetically modified crop plants into wild relatives is assessed for a new pest resistance gene in a new crop. This review summarizes the evidence for safety of crops containing Cry proteins for humans, livestock, and other non-target organisms.

## Introduction

The insecticidal activity of *Bacillus thuringiensis* (*Bt*) was discovered in 1901 in Japan, where the bacterium was isolated from infected silkworms, and was later (1911) rediscovered in Germany in infected flour moth chrysalids (reviewed in Sanchis, 2011). For over 50 years, *Bt* strains and their insecticidal proteins have been used as commercial biological pesticides (Betz et al., 2000; Sanchis, 2011). The first U.S. registration of a *Bt* microbial product was in 1961; by 1998, there were approximately 180 products registered in the U.S. Environmental Protection Agency (EPA, 1998a,b). There are reported to be over 120 microbial products in the European Union Hammond and Koch, 2012) and approximately 276 *Bt* microbial formulations registered in China (Huang et al., 2007). In China, 10s of 1000s of tons of *Bt* microbial formulations are applied to food crops, forests, and potable water, the latter as a means of controlling mosquitoes and other insect vectors of human disease (WHO/IPCS, 1999; Ziwen, 2010). The extensive use of *Bt* microbial pesticides worldwide is likely due to their specificity against a limited number of target insect species that greatly limits the potential for impacts to beneficial and non-target organisms (NTOs; Receptor-Mediated Selectivity of *Bt* Cry Proteins) and lack of environmental persistence of Cry proteins (WHO/IPCS, 1999; Betz et al., 2000; OECD, 2007; Federici and Siegel, 2008).

Because of the efficacy, safety to humans and NTOs, and favorable environmental persistence profile of *Bt* microbial formulations, the active proteins in these formulations have been isolated and optimized for expression in plants to make genetically modified (GM) crops that are resistant to target insects. The adoption by farmers of these *Bt* protein–containing crops has been dramatic, with 75.9 million hectares of *Bt* crops planted in 2013 representing over 40% of all GM crops planted that year (James, 2013). The large-scale adoption of the *Bt* crops can be attributed to increased crop productivity and reduced need for chemical pesticides which result in a reduced environmental footprint for agriculture.

This review summarizes primarily what is known about food safety, however, as part of this review the mechanism of action, taxonomic specificity, and environmental safety of *Bt* proteins particularly Cry proteins has been included. The large amount of safety information that has been generated on both *Bt* microbial insecticides and GM crops that produce specific *Bt* proteins is discussed, and technical assessments of the small number of studies that have raised concerns about the safety of *Bt* Cry proteins are provided. The many benefits of *Bt* crops beyond insect control *per se* are also highlighted.

## Mechanism of Action of *Bt* Cry Protein Insecticidal Activity

*Bt* is a Gram-positive, aerobic bacterium found in a variety of outdoor environments (Federici and Siegel, 2008). When nutrients or oxygen are insufficient for vegetative growth, the bacterium sporulates, producing a spore, and a parasporal body containing one or more insecticidal crystal proteins (Federici and Siegel, 2008). Three main types of insecticidal proteins are known to be produced by *Bt* microbes: Cry (for crystal) proteins, Cyt (for cytolytic) proteins, and toxins that are produced and secreted during vegetative growth. The diversity within and among these protein classes provides activity against a range of larval insects (van Frankenhuyzen and Nystrom, 2002; Crickmore et al., 2014). Cry proteins are active against lepidopteran insects and some variants are also active against dipteran insects, coleopteran insects, or nematodes. Cyt proteins are toxic to mosquito and black fly larvae and a few beetle species (Soberón et al., 2013). VIP proteins, which are produced and secreted during the vegetative growth stage, have activity against coleopteran and lepidopteran insect species. Although each of these components contributes to the overall insecticidal activity of microbial *Bt* products, the Cry proteins are considered the most important component of commercial *Bt* formulations. It is for this reason that the majority of commercially available GM crops with insecticidal activity have been developed to express one or more *Bt* Cry proteins, which are the focus of this review.

The mode of action of Cry proteins has been extensively studied and reviewed (WHO/IPCS, 1999; Betz et al., 2000; Siegel, 2001; Bravo et al., 2007; OECD, 2007; Federici and Siegel, 2008; Soberón et al., 2010). Unlike most chemical pesticides, which are contact insecticides, Cry proteins are effective only when ingested by the insect. The Cry proteins are approximately 70–140 kDa (Höfte and Whiteley, 1989). Within the alkaline conditions in the Lepidopteran insect gastrointestinal (GI) tract, proteases cleave the protoxin (inactive) form into an active toxic/toxin form. The activated Cry molecules typically bind to specific receptors on mid-GI-tract epithelial cells of target larval insects and then oligomerize, forming pores consisting of four to six Cry molecules each in cellular membranes. These pores enable excess cations to enter the cell, causing osmotic imbalance. The affected midgut cells take in water, swell, and lyse, eventually resulting in death of the insect (Federici and Siegel, 2008; Soberón et al., 2010). Receptors to which Cry proteins bind in insects include cadherin-like glycoproteins, which appear to be a primary receptor for some Cry proteins, and glycosylphosphatidyl-inositol (GPI) membrane anchored receptors such as aminopeptidase N or alkaline phosphatase (Soberón et al., 2010). There is also compelling evidence for ABC transporters serving as receptors for Cry proteins in several lepidopteran species (Gahan et al., 2010; Baxter et al., 2011; Atsumi et al., 2012). Receptor binding is not sufficient to cause toxicity to the insect, as oligomerization is required for pore formation (Federici and Siegel, 2008; Soberón et al., 2010). The many permutations of species-specific insect GI-tract proteases, receptors, and tendencies of each Cry protein to oligomerize in a specific situation result in the biological specificity that provides targeted insecticidal activity and safety to NTOs of *Bt* sprays and GM crops that produce *Bt* proteins.

### Receptor-Mediated Selectivity of *Bt* Cry Proteins

An essential component of the highly selective insecticidal properties of most Cry proteins is the requirement that the toxin interact with one or more specific receptors. Many studies have demonstrated that the GI-tract epithelial surface of non-target insects and mammals, including humans, lack specific high-affinity Cry protein receptors (Sacchi et al., 1986; Wolfersberger et al., 1986; Hofmann et al., 1988a,b; Van Rie et al., 1989, 1990; Noteborn et al., 1995; Lambert et al., 1996; Mendelsohn et al., 2003; Griffitts et al., 2005; Shimada et al., 2006; OECD, 2007; Vachon et al., 2012). The absence of high-affinity binding in mammals may be due in part to the absence of BL2, a glycosylating enzyme present in the GI-tract cells of invertebrates. This enzyme produces specific sugar residues that facilitate recognition and binding by Cry proteins to the aminopeptidase N and alkaline phosphatase receptors (Federici and Siegel, 2008; Soberón et al., 2010).

Experiments with surface plasmon resonance assays indicate that binding to the cadherin receptor occurs at nM concentrations (Sacchi et al., 1986; Hofmann et al., 1988a; Soberón et al., 2010), which indicates relatively high affinity, and is a characteristic of a specific receptor-ligand relationship. The fact that insecticidal LD_50_ is typically in the low ng/insect larva provides additional evidence about the potency of Cry proteins in susceptible insects that links Cry proteins and insect-specific receptors (Federici and Siegel, 2008). Specificity of Cry protein binding was shown with gut cell brush-border membrane vesicle (BBMV) binding studies. In these experiments the binding of a labeled Cry protein, which is active against *Manduca sexta* (Lepidoptera) larvae, could be displaced by an unlabeled excess of the same Cry protein or other Cry proteins active against *M. sexta,* but not by Cry proteins with activity against dipteran or coleopteran larvae (Hofmann et al., 1988b).

Extensive evidence has been accumulated on the mechanism of action for *Bt* microbials, and Cry proteins in particular, and resulted in a generally accepted model wherein Cry proteins bind to specific high-affinity receptors in the gut of target larval insects, oligomerize, and form pores in the cellular membranes of the gut. The result of pore formation is osmotic shock, cell lysis, and eventual death of gut epithelial cells. Cadherin-like glycoproteins were mentioned above as receptors, but aminopeptidase N, ABC transporters, and alkaline phosphatase have also been identified as Cry protein receptors, which enable pore formation in target organisms. BL2 is a glycosylating enzyme found in target insect pests which is responsible for producing the specific sugar residues that facilitate recognition and binding by Cry proteins to the aminopeptidase N and alkaline phosphatase receptors (Federici and Siegel, 2008; Hammond and Koch, 2012). The presence of this protein in insect species appears to contribute to the taxonomic specificity of Cry proteins currently in *Bt* crops. The absence of this protein is believed to be a reason why Cry proteins fail to exhibit toxic effects in mammals and other non-insect species.

## History of Safety Use of *Bt* Microbial Products in Agriculture

There is an extensive history of safety for human consumption of Cry proteins. *Bt* microbial pesticides have been used to control mosquito larvae in drinking water held in outdoor storage facilities (WHO/IPCS, 1999; Bravo et al., 2007) and to control insect pests on organically grown vegetables (WHO/IPCS, 1999; Federici and Siegel, 2008). Frederiksen et al. (2006) found levels of up to 10^4^ CFUs (colony-forming units, i.e., viable *Bt* microbes) per gram of plant tissue on fresh vegetables marketed in Europe. The strains found were indistinguishable from those in commercial *Bt* microbial formulations, indicating that *Bt* microbial applications were the likely source. Vegetables treated with *Bt* (i.e., broccoli, tomatoes, cucumbers, cauliflower, and lettuce) are commonly eaten raw and may be minimally washed; in such cases, people are directly consuming Cry proteins and *Bt* spores with no apparent adverse effects (Federici and Siegel, 2008).

Regulatory agencies have acknowledged the history of safe consumption of Cry proteins. For example, the U.S. EPA (1998b) stated, “The use patterns for *Bacillus thuringiensis* may result in dietary exposure with possible residues of the bacterial spores on raw agricultural commodities. However, in the absence of any toxicological concerns, risk from the consumption of treated commodities is not expected for both the general population and infants and children.” Similarly, WHO/IPCS (1999) noted that “*Bt* has not been reported to cause adverse effects on human health when present in drinking-water or food.” The lack of adverse health effects is further supported by an early test of human safety, where no adverse effects were reported when volunteers were exposed to large doses of *Bt* spores: oral ingestion of 10^10^ *Bt* spores for 5 days or inhaled 10^9^ *Bt* spores (Siegel and Shadduck, 1990) and oral ingestion of *Bt* spores at a dose of 1000 mg/day or inhalation of *Bt* spores at a dose of 100 mg/day for 5 days (Siegel, 2001). Because of their exceptional safety profile, *Bt* microbials have historically been exempted from the requirement for a numerical tolerance (maximum residue level) in countries where they have been registered (OECD, 2007), meaning that there is no withdrawal time required for consumption of crops sprayed with *Bt* microbials. Thus, the safety of *Bt* microbials, including the Cry proteins, has been well established through decades of use in various agricultural applications around the world as recognized by international health agencies (WHO/FAO, OECD, and numerous regulatory agencies).

### Human Dietary Exposure Assessment for *Bt* Microbial Products

The potential human dietary exposure to Cry proteins from application of commercial *Bt* microbial pesticide formulations was recently estimated (Hammond and Koch, 2012; **Box 1**). The adult intake of Cry protein from consumption of uncooked broccoli (heads) was estimated to be 1 μg/kg body weight for chronic consumption [5]. This estimated exposure is several orders of magnitude higher than a previous estimate of chronic dietary intake of functionally active Cry protein from food derived from GM maize expressing a *Bt* toxin Cry1Ab from a recombinant gene inserted into the plant genome (∼0.008 μg/kg body weight; Hammond and Jez, 2011; See Impact of Heat on Stability; **Box 2**).

**Box1| Assumptions underlying estimation of human dietary exposure to Cry proteins from *Bt* microbial products (Hammond and Koch, 2012).**

(1) The *Bt* microbial formulation was applied to broccoli shortly before harvest.
(2) The commercial application rate for the *Bt* microbial formulation followed the label directions from the supplier (up to 32 oz/acre).
(3) The *Bt* microbial formulation contained ∼10% Cry protein (w/w) and was applied once.
(4) 10% of the applied *Bt* microbial formulation was deposited on broccoli heads.
(5) Only broccoli heads were consumed.
(6) Broccoli consumption was set to the chronic dietary intake of broccoli in the U.S. (8.2 gm/capita/day, Latté et al., 2011).
(7) The broccoli was consumed raw.

**Box2| Assumptions underlying estimation of human chronic dietary exposure to Cry1Ab protein from *Bt* maize (Hammond and Cockburn, 2008; Hammond and Jez, 2011).**

(1) Corn is consumed at 50th percentile for adults in the UK (16 g/day).
(2) Level of Cry1Ab protein in unprocessed Cry1Ab maize grain is 0.3 ppm (μg/g).
(3) Average adult body weight is 60 kg.
(4) All maize consumed is Cry1Ab maize.
(5) Thermal processing reduces functional activity by at least two orders of magnitude.

## The Use of *Bt* Cry Proteins in GM Crops

Although *Bt* microbial preparations are safe and efficacious, they are limited in their duration of effectiveness because they can be washed off the plant (e.g., by rain) or inactivated by sunlight within days after application (Federici and Siegel, 2008), and they require considerable water, heat, and feedstock to produce, and must be manually applied, either by hand sprayer on small plots or by machine if applied to large tracts. These limitations have been addressed through the introduction of GM crops containing one or more genes encoding one or more *Bt* proteins, which are produced by the plant throughout the growing season. Dietary exposure to GM derived *Bt* proteins may be equal to or less than that of *Bt* microbial products (See Impact of Heat on Stability). Another advantage of GM crop products expressing *Bt* proteins is that only the specific *Bt* protein(s) of interest, typically Cry proteins, are produced in the crop, possibly decreasing the spectrum of activity compared to *Bt* microbial formulations (Hammond and Koch, 2012).

Most of the commercially approved *Bt* crops are corn and cotton (**Table 1**). Recently, *Bt* soybean varieties expressing the *cry1Ac* gene and Cry1Ac and Cry1F have been approved for commercial use in Latin America to control lepidopteran pests (CTNBio, 2010; EPA, 2010b, 2014), and *Bt* rice varieties with the *cry1Ab* and *Cry1Ac* genes has been developed in China but initial approval expired in 2014 (ISAAA, 2015).

**Table 1:** ***Bt* Cry proteins in GM crops authorized for cultivation in one or more countries**.

Protein	Insect type controlled	Crop species approved^a^	Examples of products^b^(Registrant)
Cry1Ab	Lepidoptera	Maize	YieldGard (Monsanto) Agrisure CB/LL (Syngenta)
Cry1Ac	Lepidoptera	Cotton	Bollgard (Monsanto)
		Maize^c^	*Bt* Xtra (Monsanto)
		Soy	Intacta Roundup Ready 2 Pro (Monsanto)
		Brinjal	BARI *Bt* Begun-1, -2, -3, -4 (MAHYCO)
Cry1A.105 + Cry2Ab2	Lepidoptera	Maize	Genuity VT Double Pro (Monsanto)
Cry1Ac + Cry2Ab2	Lepidoptera	Cotton	Bollgard II (Monsanto)
Cry1Ac + Cry1F	Lepidoptera	Cotton	WideStrike (Dow)
		Soy	DAS-81419-2 (Dow)
Cry1Fa2	Lepidoptera	Maize	Herculex I (Dow)
Cry1Ab + Cry2Ae	Lepidoptera	Cotton	TwinLink (Bayer)
mCry3A	Coleoptera	Maize	Agrisure RW (Syngenta)
Cry3Bb1	Coleoptera	Maize	YieldGard Rootworm RW (Monsanto)
eCry3.1Ab^d^	Multiple	Maize	Agrisure Duracade (Syngenta)
Cry34Ab1 + Cry35Ab1	Coleoptera	Maize	Herculex RW (Dow and DuPont)

*Data from*http://www.isaaa.org/gmapprovaldatabase/

*^a^Approval does not mean that crops were planted commercially or are still being planted.*

*^b^Not all products and combinations are listed.*

*^c^With pinII (protease inhibitor) from Solanum tuberosum.*

*^d^Chimeric Cry3A–Cry1Ab protein.*

Another type of modification that has been used when developing a *Bt* Cry-containing GM crop is swapping portions or whole domains from one Cry protein with portions or whole domains from another Cry protein (Höfte and Whiteley, 1989; Nakamura et al., 1990; Ge et al., 1991; Honée et al., 1991). Domain swapping has been shown to be an effective way to change the spectrum of activity of a native Cry protein to include a new target pest. Examples of proteins that have undergone domain swapping for this purpose are Cry1A.105 and Cry1Ab/Ac; the former is one of a series of modified proteins that exhibit improved activity toward fall armyworm larvae but otherwise retained specificity toward other target lepidopteran pest species (Malvar et al., 2004). Such domain exchanges are possible, in part, because the tertiary structures of these Cry domains are highly conserved (Li et al., 1991; Grochulski et al., 1995; Derbyshire et al., 2001; Galitsky et al., 2001). As another example, the Cry1Fa-like protein in the *Bt* microbial Lepinox (construct 11724) contains the core Cry1Fa insecticidal protein moiety fused to a Cry1Ac C-terminal protoxin moiety (Baum et al., 1999). This modified protein exhibited improved expression in *Bt* but retained the insecticidal specificity of the native Cry1Fa protein (Gilmer and Baum, 1999). Recently, the chimeric eCry3.1Ab insecticidal protein was developed to provide protection against root feeding damage caused by the western corn rootworm, northern corn rootworm, and Mexican corn rootworm. The eCry3.1Ab protein is composed of domains I, II, and a portion of domain III of the native Cry3A protein and the C-terminus of the Cry1Ab protein (Walters et al., 2010; EPA, 2013).

There are several important, well-established examples that demonstrate that small changes in *Bt* amino acid sequence do not change the safety profile for NTOs. Regulatory authorities have required functional studies with sensitive insect bioassays to demonstrate that these small changes do not impact biological activity. If these assays indicate biological activity equivalence of the two protein forms, any other properties of the proteins are considered to be equivalent as well. Consequently, it is not necessary to repeat all of the assays performed for the original safety assessment; regulators will consider “bridging” to the form of the protein that was used for the environmental safety testing. A well-established example of this approach was used to bridge the existing safety assessment performed for the Cry3Bb1 protein produced by MON863 maize to support the approval of MON88017 maize, which also produces a variant of the Cry3Bb1 protein. The Cry3Bb1 protein expressed in MON88017 maize is functionally and physicochemically similar to that expressed in MON863 maize. The proteins differ by only 1 amino acid out of 653 (99.8% homology) and are expressed at comparable levels in the plant. To test for functional equivalence, sensitive insect diet incorporation bioassays were performed with appropriate insect models (i.e., Colorado potato beetle and western corn rootworm) to estimate LC_50_ values. The results were similar for both Cry3Bb1 variants (EPA, 2010c). Another example is the form of the Cry2Ab2 protein produced by MON15985 cotton and MON89034 maize. These two forms of the Cry2Ab2 protein differ by a few amino acids on the N-terminus of the proteins and reflect the introduction of a chloroplast transit peptide (EPA, 2010a). Despite the addition of these amino acids, there was no impact on biological activity in a sensitive insect bioassay (EPA, 2010a).

Critics have raised safety concerns about modifications that have been made to *Bt* Cry proteins introduced into GM crops. They consider the modified Cry proteins as not “natural,” arguing that their safety is unknown and they should be subjected to chronic animal testing as is done for small-molecule chemical pesticides (Séralini et al., 2011). There are two problems with this rationale. First, whether or not a substance is “natural” has very little to do with its intrinsic safety [e.g., α-latrotoxin occurs naturally in spiders of the genus *Latrodectus*, and is quite lethal in very small amounts – the LD_50_ in mice is 20–40 μg/kg (Ushkaryov et al., 2004)]. Rather, the focus should be on whether the modifications fundamentally alter the structure and functional properties of the protein to a degree that it affects the safety of these proteins (See History of Safe Use and Bioinformatics). Thus, despite the changes introduced, the extensive history of safe human consumption of native Cry proteins can be applied to the safety assessment of these modified proteins (Hammond et al., 2013). Second, *Bt* Cry proteins in either their microbial or plant-incorporated protectant (i.e., GM crops) form are classified as biopesticides by the U.S. EPA (2015). This is an important designation because biopesticides are generally considered be inherently less toxic and have a narrower spectrum of activity (i.e., affect only the target pest and closely related organisms) than conventional pesticides (EPA, 2015). Accordingly, the current testing paradigm is sufficient for detecting potential hazards associated with GM crops; especially, when considered with the Weight of Evidence accumulated from the analyses subsequently detailed in Section “Food Safety Assessment of Cry Proteins Introduced into *Bt* Crops” and its subsections.

## Food Safety Assessment of Cry Proteins Introduced into *Bt* Crops

Several International Life Sciences Institute (ILSI) task forces have examined and provided science-based recommendations for the safety assessment of crops derived from agricultural biotechnology. Principles for the assessment of proteins used in agricultural biotechnology were described in detail by Delaney et al. (2008) and revisited and expanded by Hammond et al. (2013). Both describe a two-tiered approach of hazard identification followed by hazard characterization appropriate to the identified hazards, if any. Regulatory guidance on the assessment of insecticidal proteins has been given by both national and international authorities (Mendelsohn et al., 2003; Codex Alimentarius, 2009; EFSA, 2011).

### History of Safe Use and Bioinformatics

One of the steps in initial (“Tier I”) evaluation of a protein is to examine its history of safe use (HOSU). As discussed in Section “The Use of *Bt* Cry Proteins in GM Crops,” some *Bt* proteins are modified from their native form for use in GM crops; thus, it is important to consider whether the HOSU of one protein can be applied to related proteins (Hammond et al., 2013).

Although there are seemingly an unlimited number of changes that could be made to improve protein function, one estimate indicates that only 0.01–0.5% of random amino acid sequence modifications are beneficial (Bloom and Arnold, 2009). Looking further into the types of changes that a protein can undergo can help explain this estimate. Substitution of one amino acid residue with another one of similar size and properties might have little effect; on the other hand, substitutions that alter the three-dimensional structure will likely have major deleterious consequences on protein functionality. Modifications that alter the ability of the protein to fold properly are often deleterious because they reduce or eliminate the functional activity. Although radical changes are possible, amino acid sequence modifications introduced in engineered proteins are normally designed to enhance function with minimal disruption of structure. By retaining the three-dimensional structure of the naturally occurring protein, the general functional characteristics are also preserved in the engineered protein (Behe et al., 1991; Lattman and Rose, 1993; Rose and Creamer, 1994). This is also true for most evolutionary changes because the structure and function of an essential protein must be conserved (Illergård et al., 2009).

When *in vitro* engineering introduces minor changes to the structure or function of a protein, there is little reason to suggest that the engineered protein will become toxic. This has been the case for evolutionary changes within protein families such as those used as food processing enzymes. These changes have not resulted in the enzymes becoming toxic to humans (Pariza and Cook, 2010). As an example of how difficult it is for a set of specific mutations to convert a non-toxic protein into one that is toxic, Pariza and Cook (2010) calculated that the probability that nine amino acid substitutions could make a non-toxic protein into a known protein toxin was only 1 in 2 × 10^11^. For context, this is similar to selecting a specific drop of water in one of five Olympic-sized swimming pools on the first attempt. As illustrated by the specificity of Cry proteins for their target insects (See Receptor-Mediated Selectivity of *Bt* Cry Proteins), the effects of protein toxins in susceptible organisms generally depend on recognition of specific molecular targets (Rappuoli and Montecucco, 1997).

In addition to considering the HOSU of related proteins, performing bioinformatic analyses of protein sequence and structure can provide valuable information on the safety of a modified Cry protein or other introduced protein. A bioinformatics evaluation is performed as one of the initial steps in the Tier 1 safety assessment (Delaney et al., 2008). These comparisons of the candidate protein to sequence and structure databases are used to determine whether the protein of interest is similar to any known toxic protein. Analyses of phylogenetic relationships between the candidate protein and others are also useful (Codex Alimentarius, 2009; EFSA, 2010e, 2011; Hammond et al., 2013) because high similarity may indicate a conserved function (e.g., Mills et al., 2004).

### Human Dietary Exposure Assessment for Cry Proteins in *Bt* Crops

The information gathered from the safety assessments described throughout this manuscript identifies if there is a potential hazard associated with the Cry proteins and are necessary for performing dietary risk assessments. However, a determination of exposure is also required because risk is a function of both hazard and exposure (Faustman and Omenn, 2008).

An estimate of the maximum potential exposure to a Cry protein through the diet can be derived by determining the protein expression level in the grain and the amount of grain consumed. Such values are highly conservative estimates of dietary exposure because they do not account for the lack of stability of the protein during digestion, they do not consider its response to heat or food processing conditions, they ignore that commodity crops are a blend of multiple varieties (i.e., all the crop consumed is presumed to be the variety in question), and they often utilize very high consumption levels (i.e., 97.5th percentile “Eater-Only” values). The harsh conditions encountered by dietary proteins in the GI lumen and during food production processes significantly reduce their stability as detailed below.

#### Impact of Protein Digestibility

Most ingested dietary proteins undergo hydrolytic digestion and/or degradation (Delaney et al., 2008). To approximate the effects of protein exposure to conditions in the mammalian GI tract, a validated *in vitro* assay to assess the potential stability of proteins to pepsin digestion has been developed. This reliable and reproducible assay uses a fixed pepsin:protein ratio and low pH (pH 1.2 and 2.0; Thomas et al., 2004). Cry proteins are readily degraded in this assay (EPA, 2001; Okunuki et al., 2002; Herman et al., 2003; Thomas et al., 2004; Cao et al., 2010; Guimaraes et al., 2010). Under conditions of higher pH and lower ratios of pepsin to Cry protein, Cry1Ab protein is more slowly degraded, as is expected since pepsin becomes less active at a greater pH (Guimaraes et al., 2010). Although Guimaraes et al. (2010) suggest that the current low-pH test may need to be revisited, Ofori-Anti et al. (2008) reported that, as anticipated based on classic enzymology, varying pH, and pepsin concentration had only small effects on digestion of proteins of intermediate stability to pepsin and no effects on proteins that are either stable in the presence of pepsin or rapidly digested by pepsin.

In pigs and calves, Cry protein fragments are detectable but are progressively reduced in size as they travel down the GI tract. None were detected in the liver, spleen, or lymph nodes (Chowdhury et al., 2003a,b) indicating they were too large to be systemically absorbed from the GI tract. It has been suggested that transgenic nucleic acids and proteins from GM crops are handled in the gut like their conventional counterparts, with no evidence for systemic absorption of intact proteins or genes (Światkiewicz et al., 2009; Walsh et al., 2012a,b; Sieradzki et al., 2013). It is notable that farm animals generally have a much higher proportion of maize in their diets than humans; for example, in the case of broiler chickens, maize accounts for approximately 65% of their diet throughout their productive lifetime. In addition, maize fed to animals is generally not processed, other than grinding. Human dietary exposure to Cry proteins is much lower than that of farm animals owing to the lower percentage of maize in the diet and the processing (e.g., cooking) used to prepare most human food containing maize. As described in the following Section “Impact of Heat on Stability,” thermal processing denatures Cry proteins, causing them to lose insecticidal activity (**Table 2**) and making them more susceptible to protease degradation (Okunuki et al., 2002; Herman et al., 2006). Recalling that risk is a function of both hazard and exposure, the lower exposure encountered by humans puts them at lower risk than animals. This is particularly noteworthy when one considers that the animals did not demonstrate reliable signs of toxicity (i.e., no hazard was identified) following the consumption of GM crops, despite considerably higher dietary exposure.

**Table 2:** **Impact of heating and food processing on Cry proteins**.

Introduced protein	*In vitro* heat	Functional activity/ Immunodetectability	Reference
Cry1Ab	80∘C; 10 min	Insect bioassay – loss of activity	de Luis et al. (2009)
Cry1F	75–90∘C; 30 min	Insect bioassay – loss of activity	EFSA (2005c)
Cry9C	90∘C; 10 min	Insect bioassay – No loss of activity	de Luis et al. (2009)
Cry34Ab1, Cry35Ab1	60–90∘C; 30 min	Insect bioassay – loss of activity	EFSA (2007)
eCry3.lAb	95∘C; 30 min	Insect bioassay – loss of activity	EPA (2013)
Cry1A.105	204∘C; 20 min	No immunodetectability	EPA (2010a)
Cry2Ab2	204∘C; 20 min	No immunodetectability	EPA (2010a)
Cry1Ac	Not stated in Biopesticides Registration Action Document	No immunodetectability	EPA (2010b)

*After Hammond et al. (2013)*

#### Impact of Heat on Stability

Prior to human consumption, Cry proteins in GM crops typically undergo some sort of thermal processing, which must be considered in the toxicological safety assessment because it can significantly reduce the dietary exposure to functionally active Cry protein. Temperature increases, pH variation, and physical disruption can overcome the forces that keep a protein folded properly (Creighton, 1993) and cause denaturation, i.e., changes in protein secondary, tertiary, or quaternary structure. Because of the change in three-dimensional structure, denatured polypeptides lose functional activity, including the ability to specifically bind receptors or other compounds.

A number of Cry proteins have been subjected to *in vitro* heat stability studies under conditions similar to those used for human food processing (Hammond and Jez, 2011; **Table 2**). All Cry proteins tested except Cry9C, which was modified by its developer for enhanced stability (Center for Environmental Risk Assessment, 2001), lost insecticidal activity or immunodetectability after processing (**Table 2**). The impact of food processing on the functional activity of introduced proteins is relevant because for cooked food, it results in safety margins that are conservative and an overestimate of actual exposure (i.e., the dietary exposure estimates do not typically include the effects of processing on exposure). The European Food Safety Authority (EFSA GMO Panel Working Group on Animal Feeding Trials, 2008) agrees that the effects of food processing may result, “…in over-estimated exposure levels and even larger margins of safety for man.” It has been estimated that when using heat denaturation studies the levels of functionally active protein introduced into a GM crop can be reduced by approximately two orders of magnitude (100-fold) when the crop is processed (e.g., by cooking; Hammond and Jez, 2011).

For *Bt* maize containing different Cry proteins, the levels of Cry proteins in grain were approximately 0.3–0.7 ppm for Cry1Ab and approximately 15–115 ppm for other Cry proteins (Hammond and Cockburn, 2008). Using the conservative assumptions detailed in Section “Human Dietary Exposure Assessment for Cry Proteins in *Bt* Crops” for human dietary exposures (Hammond and Jez, 2011), the potential human dietary intake of functionally active Cry protein from *Bt* maize could range from 0.008 to 2 μg/kg body weight [BW]/day (Hammond and Koch, 2012). Even these values may overestimate exposure, as the calculation for exposure to Cry1Ab *Bt* protein in Hammond and Jez (2011) of 0.008 μg/kg BW appears to reflect only a 10-fold reduction in activity caused by cooking (vs. the intended 100-fold reduction). Thus, dietary exposure from *Bt* crops may be similar to or even less than dietary exposure from vegetables treated with *Bt* microbial formulations shortly before harvest (estimated in Section “Human Dietary Exposure Assessment for *Bt* Microbial Products”).

Clearly, digestion and food processing greatly limit the dietary exposure of any proteins consumed orally whether transgenic or endogenous. Nonetheless, companies seeking registration of biotech crops often use the highly conservative approach of estimating exposure by determining the protein expression in the grain and multiplying it by the amount of grain consumed. This obviously overestimates exposure for the reasons detailed above. Despite this conservative approach in estimating dietary exposure (i.e., over-estimating dietary exposure), it has still been possible to achieve very large margins of exposure between the levels of Cry proteins safely administered to animals and conservative estimates of human intake (Juberg et al., 2009).

### Allergy Safety Assessment of Cry Proteins

The allergy assessment of a GM crop is designed to identify potential allergenic risks associated with an introduced protein (Codex Alimentarius, 2009). A weight-of-evidence approach is taken to assess the allergenic potential of the introduced protein. This assessment includes determining whether the source organism of the introduced protein is allergenic, an extensive bioinformatics assessment to determine if the introduced protein is similar to known allergens, and determining the level of exposure of the introduced protein in the GM crop, including determining the stability of the protein to digestive enzymes (Goodman et al., 2005; Lehrer and Bannon, 2005; Bannon and Martino-Catt, 2007).

*Bacillus thuringiensis* microbials are not considered a human allergen, because despite the extensive use as a biopesticide over the last several decades, there has been only one report of allergic reactions in workers who manufacture or apply *Bt* microbials (Siegel, 2001; Federici and Siegel, 2008). However, this single allergic reaction was attributed to bacterial proteins in the *Bt* microbial formulation other than the Cry proteins (Siegel, 2001; Federici and Siegel, 2008). None of the *Bt* Cry proteins used to develop GM crops have had any sequence similarity to known human allergens using any of the bioinformatics thresholds for amino acid similarity recommended by international guidance (Codex Alimentarius, 2009; Silvanovich et al., 2009).

Another assessment of allergenic potential of the inserted protein is to determine the exposure to the protein. This is relevant to the allergenicity assessment because it is generally accepted that increased exposure to a protein increases the possibility that the protein could become an allergen. Exposure is assessed by measuring the protein abundance in the grain (See Impact of Heat on Stability) and the stability of the protein in the presence of pepsin (See Impact of Protein Digestibility). Unlike the toxicological safety assessment, the heat stability assessment of the protein does not provide useful information to the allergy safety assessment because an allergen does not need to be in a functional state to cause an allergic reaction (Privalle et al., 2011). Although pepsin resistance affects the potential exposure to the introduced protein, it is important to point out there is evidence that some allergens are present in high levels in food crops and/or resistant to digestion, but there are also non-allergenic proteins that have these same properties (Fu et al., 2002). For this reason, it is important to recognize that these analyses are not predictive of allergenic potential, but instead should be considered as part of the weight-of-evidence approach. Taken together, the weight-of-evidence approach used to assess the Cry proteins present in *Bt* crops suggests the allergenic potential of these proteins is low and that they present little allergenic risk.

### Acute and Short-Term Mammalian Toxicology Testing of *Bt* Cry Proteins

In the paradigms outlined by Delaney et al. (2008) and Hammond et al. (2013) and introduced earlier in this manuscript, Tier II studies such as toxicology testing are only warranted when a potential hazard has been identified in Tier I. Acute toxicology testing was originally warranted because of the insecticidal (i.e., toxic) acute mode of action of Cry proteins. As described in subsequent sections, neither short- nor long-term toxicology testing has revealed any concerns about the safety of *Bt* proteins for human or animal consumption.

In general, there are fundamental biological properties of proteins that greatly limit their potential to produce chronic toxic effects when ingested (Delaney et al., 2008; Hammond et al., 2013). These properties are discussed in detail in Hammond et al. (2013) and some of the key points are summarized here:

(1) Protein macromolecules are degraded by the digestive enzymes of the GI tract into small peptides and individual amino acids to facilitate absorption. Furthermore, it is generally known that there is an inverse relationship between molecule size and absorption [smaller molecules are more readily absorbed intact than larger ones (Gardner, 1988)]. Consequently, the potential for absorption of an intact protein from the GI tract is much lower than a low molecular-weight chemical.
(2) Multiple and redundant barriers restrict the movement of intact proteins into cells after dietary consumption (Kier and Petrick, 2008). With the exception of nursing newborns, a primary barrier in humans is the plasma membrane of GI tract epithelium. These membranes contain receptors and transporters that enable the intact uptake of specific proteins, but are otherwise impermeable to exogenous proteins (Gardner, 1988). Repeated failures to develop orally administered protein-based therapeutics (O’Hagan et al., 1988; Shah et al., 2002; Goldberg and Gomez-Orellana, 2003; Hamman et al., 2005) demonstrate the effectiveness of these barriers.
(3) The ingestion of proteins has not been shown to be carcinogenic, teratogenic, or mutagenic (Pariza and Foster, 1983; EPA, 2000; Pariza and Johnson, 2001), and the proteins introduced to date into GM crops are not considered to be toxic based on their known biochemical function and on the results obtained from bioinformatics searches.
(4) Based on the biochemical functions of known protein toxins, toxicity is usually manifest after acute or short-term dietary exposure. Additionally, there is no example of a protein that bioaccumulates with chronic dietary intake. Instead, proteins are degraded to amino acids that are reassembled into new proteins. There are no storage proteins in animals and humans; rather, energy is stored as adipose tissue, which is biochemically distinct from protein. Thus, chronic testing is unlikely to add meaningfully to the safety assessment of GM crops.

Considering the first two points above, and that Cry proteins are readily digested (See Impact of Protein Digestibility) and can be denatured and inactivated during normal food processing (See Impact of Heat on Stability), human dietary exposure to functionally active dietary proteins, including Cry proteins, is likely to be negligible.

The U.S. EPA (2000) requires the use of high-dose (gram/kg BW) acute testing for Cry proteins where feasible because they act through an acute mode of action to kill insect pests (Sjoblad et al., 1992; Delaney et al., 2008). While acute oral toxicity studies with proteins from GM crops have fallen out of favor in some world areas (e.g., the EU), they are still commonly conducted as part of the safety assessment in other world areas (e.g., China). Current acute oral toxicity study designs may be adapted from relevant guidance in OECD Guidelines for the Testing of Chemicals (i.e., OECD Test Guidelines 420, 423, and 425). It has not always been possible to achieve gram/kg dosage levels because of the limited solubility of some Cry proteins in dosing vehicles (McClintock et al., 1995; Betz et al., 2000); nevertheless, the dosages administered to date have still been many orders of magnitude higher than any dose that humans might potentially encounter in the diet. Recently, EFSA (2011) stated that they may require a repeat-dose 28-day toxicology study when it considers the available safety information on the introduced protein to be insufficient. What this means in practice has not yet been determined for Cry proteins, but given the substantial HOSU of Cry proteins and the existing weight of evidence for approved traits, there should be sufficient scientific evidence on the safety of such proteins to preclude the need for conducting a 28-day study. This is particularly important when one considers the scientific community’s obligation to reduce, replace, and refine animal studies when scientifically feasible, and the fact that such studies are unethical when they provide little additional information of value to risk assessment (See Whole Food Animal Feeding Studies with *Bt* Crops).

**Table 3** presents the no-observed-adverse-effect levels (NOAEL) for a variety of Cry proteins and *Bt* microbials fed to mice in acute toxicity studies at doses 1000s to 1,000,000s of times higher than the doses that are acutely toxic to the target insects (Hammond and Cockburn, 2008). A good way to illustrate this disparity is to consider an extreme example. To attain the same acute dose of Cry1Ab protein as that administered to mice with no adverse effects (4000 mg/kg/day; **Table 3**), an adult human would have to consume approximately 900,000 kg of uncooked *Bt* maize grain in 1 day (Hammond and Cockburn, 2008). This amount of shelled corn would be the equivalent of 35 semi-truck loads. Thus, these data represent a considerable weight of evidence that Cry proteins are safe for consumption as food and feed; especially when considered with the safety of microbial *Bt* pesticides (See History of Safety Use of *Bt* Microbial Products in Agriculture); as well as bioinformatics (See History of Safe Use and Bioinformatics), *in vitro* digestibility assays (See Impact of Protein Digestibility), low dietary exposure levels (See Human Dietary Exposure Assessment for *Bt* Microbial Products), and the absence of toxicity following high dose acute studies (See Acute and Short-Term Mammalian Toxicology Testing of *Bt* Cry Proteins) with the Cry proteins tested to-date.

**Table 3:** **Acute toxicity studies in mice with Cry proteins and *Bt* microbials**.

Test substance	NOAEL^a^	Reference
Cry protein		
Cry1Ab	4000 mg/kg	Betz et al. (2000)
Cry1Ab/Cry1Ac fusion protein	5000 mg/kg	Xu et al. (2009)
Cry1A.105	2072 mg/kg	EPA (2010a)
Cry 1Ac	4200 mg/kg	Betz et al. (2000)
Cry1C	5000 mg/kg	Cao et al. (2010)
Cry2Aa	4011 mg/kg	Betz et al. (2000)
Cry2Ab	1450 mg/kg	Betz et al. (2000)
Cry2Ab2	2198 mg/kg	EPA (2010a)
Cry3A	5220 mg/kg	Betz et al. (2000)
Cry3Bb	3780 mg/kg	Betz et al. (2000)
Cry1F	576 mg/kg	EPA (2001)
Cry34Ab1	2700 mg/kg	Juberg et al. (2009)
Cry35Ab1	1850 mg/kg	Juberg et al. (2009)
eCry3.1Ab	2000 mg/kg	EPA (2013)
*Bt*microbial product		
Cry1Ac, Cry2A, Cry1C* Btk* Crymax	>10^8^ CFU^b^/rat	Betz et al. (2000)
Cry1Aa, Cry1Ac, Cry2A, Cry1Fa/1Ac *Btk* Lepinox 11724	>10^8^ CFU/rat	Betz et al. (2000)
Cry4A, Cry4B, Cry10A &11A, Cry11	>10^11^ CFU/rat	Betz et al. (2000)
Cry3Aa *Bti* Teknar	5050 mg/kg	Betz et al. (2000)

*^a^Highest dosage tested that caused no adverse effects.*

*^b^Colony-forming units.*

*After Hammond and Cockburn (2008)*.

## Assessment of Issues Raised Concerning Cry Proteins

This section will discuss in detail the technical deficiencies of some of the papers that report potential health concerns with *Bt* microbials or Cry proteins. It will also examine the reasons that, despite past practices, long-term animal testing with *Bt* crops is not scientifically beneficial or justified.

Despite the considerable history of safe consumption, and the large volume of data demonstrating the safety of both *Bt* formulations and Cry proteins, some parties still doubt the safety of these products. Occasional publications will make startling claims: for example, some claim that *Bt* toxins can increase allergenic potential, or cause hematotoxic reactions, or represent an uncharacterized risk to pregnant mothers. Yet upon closer examination, these allegations do not appear to have a strong scientific basis.

When one considers the overall demonstrated safety of Cry proteins and the lower dietary exposure to *Bt* proteins from consumption of a GM crop than microbial *Bt* formulations used in conventional and organic agriculture, claims of adverse effects exclusively following the consumption of *Bt* crops simply do not withstand closer scrutiny.

### Whole Food Animal Feeding Studies with *Bt* Crops

There is a long safety history of *Bt* microbial pesticides on agricultural crops (See History of Safety Use of *Bt* Microbial Products in Agriculture), and there is similarly a HOSU of *Bt* crops since 1996 when these crops were first introduced into commerce. Following review of relevant data in submitted dossiers from registrants, the U.S. FDA and EPA have not, to date, considered additional animal toxicology studies with whole foods (i.e., GM corn grain or soy meal) as necessary to confirm safety. Rather, they have considered the weight of evidence comprised in part by HOSU, the demonstrated safety of the trait in mammals (i.e., acute and subchronic toxicity testing results), and compositional and agronomic tests to come to address unintended effects and come to the conclusion that *Bt* crops are as safe as their conventional comparators. However, 90-day rodent subchronic feeding studies with whole foods were often required by some countries in the EU to confirm the safety of the first generation of *Bt* crops (**Table 4**). Additional repeat-dose toxicology studies that have been conducted for other purposes are also summarized in **Table 4** including reproduction and chronic studies on *Bt* crops as well as feeding studies on commercial *Bt* microbial formulations (see also Bartholomaeus et al., 2013).

**Table 4:** **Examples of repeat-dose toxicity studies with *Bt* microbial formulations, Cry proteins, and *Bt* crops**.

Test article	Dose/dietary level	Study type and test animal	Reference
*Bt*microbial formulation			
DIPEL *Bt* microbial	8400 mg/kg	90-day rat	Betz et al. (2000)
DIPEL *Bt* microbial	8400 mg/kg	2-year rat	Betz et al. (2000)
TEKNAR *Bt* microbial	4000 mg/kg	90-day rat	Betz et al. (2000)
Berliner *Bt* microbial	1000 mg/adult	5-day (human)	Betz et al. (2000)
Cry protein			
Cry34Ab1 and Cry35Ab1 (CRW)	205 mg/kg	4-week mouse	Juberg et al. (2009)
*Bt* crop			
*Bt* tomato	10% in diet^a^	90-day rat	Noteborn et al. (1995)
*Bt*/HT^b^ maize(ECB^c^/RR^d^)	11/33% in diet^a^	90-day rat	EFSA (2005d)
*Bt* /HT maize(CRW^e^/RR)	11/33% in diet^a^	90-day rat	EFSA (2005e)
*Bt* /HT maize(ECB/CRW/RR)	11/33% in diet^a^	90-day rat	EFSA (2005b)
*Bt* maize (ECB/CRW)	11/33% in diet^a^	90-day rat	EFSA (2005a)
*Bt* maize (ECB)	11/33% in diet^a^	90-day rat	Hammond et al. (2006b)
*Bt* maize (CRW)	11/33% in diet^a^	90-day rat	Hammond et al. (2006a)
*Bt* maize (ECB)	11/13% in diet^a^	90-day rat	MacKenzie et al. (2007)
*Bt* cotton	10% in diet^a^	90-day rat	Dryzga et al. (2007)
*Bt* rice	60% in diet^a^	90-day rat	Schrøder et al. (2007)
*Bt/* HT maize(CRW/Gluf^f^)	35% in diet^a^	90-day rat	Malley et al. (2007)
*Bt* /HT maize(CRW/RR)	11/33% in diet^a^	90-day rat	Healy et al. (2008)
*Bt* maize (CRW)	50/70% in diet^a^	90-day rat	He et al. (2008)
*Bt* /HT maize(ECB/CRW)	34% in diet^a^	90-day rat	Appenzeller et al. (2009)
*Bt* rice (Cry1Ab/Cry1Ac)	60% in diet^a^	90-day rat	Wang et al. (2013)
*Bt* rice (Cry1Ac)	73–82% in diet^a^	78-week rat	Zhang et al. (2014)
Multigenerational studies			
*Bt* maize (ECB)	68% in diet^a^	5-generation rat	Haryu et al. (2009)
*Bt* maize (ECB)	20% in diet^a^	3-generation rat reproduction	Kiliç and Akay (2008)

*^a^Percent (w/w) maize, rice, or cottonseed meal added to the diet.*

*^b^HT, herbicide tolerant.*

*^c^ECB, European corn borer.*

*^d^RR, Roundup Ready®; (tolerant to glyphosate herbicide).*

*^e^CRW, corn rootworm.*

*^f^Gluf, glufosinate (tolerant to glufosinate herbicide).*

To test whether GI impairment would alter the potential toxicity of Cry1Ab protein, Onose et al. (2008) used a subchronic GI impairment rat model. Rats were treated with famotidine (to reduce gastric acid secretion) and indomethacin (to damage the intestinal epithelium) and fed diets with or without Cry1Ab protein (10 ppm). Despite the expectation of less Cry1Ab protein digestion and more absorption of Cry1Ab protein into the circulatory system of the GI-impaired animals, there was no evidence of meaningful toxicological effects (changes in clinical blood parameters and histologic appearance of organs) in the Cry1Ab-dosed animals.

There have also been a few multigeneration reproduction studies carried out with animals fed *Bt* crops (**Table 4**). There were no treatment-related adverse effects seen on reproductive performance in animals fed *Bt* crops or other transgenic crops (Snell et al., 2012). While there were occasional differences observed between control and treated animals in blood parameters and organ weights, none were considered to be treatment related, consistent with the weight of evidence of numerous subchronic animal studies that have been carried out with a variety of different *Bt* crops (**Table 3**). One reproduction study (Kiliç and Akay, 2008) reported minor histologic findings in the rats fed *Bt* maize, but these findings were not consistent with the weight of evidence of many other subchronic rat studies where no evidence of treatment-related histologic changes have been reported.

EFSA GMO Panel Working Group on Animal Feeding Trials (2008) published a review article that included their assessment of a number of published toxicology studies that had been conducted on GM crops (*Bt* and non-*Bt*). According to the review, the majority of these studies showed no adverse effects. EFSA GMO Panel Working Group on Animal Feeding Trials (2008) reported that while some studies reported adverse effects in animals, deficiencies in these studies made the results uninterpretable. As a result, these studies should not be used to inform the risk assessment process for these crops. Similarly, independent researchers reviewed 24 long-term or multigenerational studies with GM crops and came to the conclusion, “…GM plants are nutritionally equivalent to their non-GM counterparts and can be safely used in food and feed” (Snell et al., 2012). The issues surrounding whole food testing of GM crops (*Bt* and non-*Bt*) have recently been examined by two groups of authors (Bartholomaeus et al., 2013; Kuiper et al., 2013). Considering the limitations of whole food testing, such as low sensitivity and difficulty in defining the test material, both groups concluded that routine whole food testing does not add meaningful information to the risk assessment of GM crops and cannot be scientifically justified.

Another recent review article evaluated the reliability of a number of the published toxicology studies carried out with GM crops and proteins in general, and Cry proteins and *Bt* crops in specific, by adapting the ToxRTool (Koch et al., 2014). The ToxRTool was originally developed to objectively assess the reliability of published toxicology studies on chemicals for the program on Registration, Evaluation, Authorization, and Restriction of Chemicals (REACH) in Europe. When its objective criteria were adapted for feeding studies and used to evaluate studies with *Bt* crops most of the studies were considered to be reliable. Of the studies determined to be reliable, none found evidence of adverse effects from the consumption of GM crops containing Cry proteins or of purified Cry proteins (Koch et al., 2014).

### Adjuvanticity

Other papers have reported that Cry proteins, present in both *Bt* microbial formulations and GM crops, stimulate IgG, IgM, and IgA antibody production following intraperitoneal (IP), intragastric (IG), intranasal (IN), or intra-rectal (IR) administration (Vázquez-Padrón et al., 1999a,b, 2000a,b; Moreno-Fierros et al., 2000).

The biological relevance of these studies to assessing potential health risks from human consumption of foods derived from *Bt* crops is limited because administration of Cry proteins by IN, IP and IR routes of exposure do not predict risks from IG or dietary intake. IN, IP, and IR routes of exposure bypass the protective barriers of the GI tract and are not particularly relevant when characterizing hazards that might be associated with a protein that will be consumed as food. However, when an IG route of exposure was used, it was necessary to include a magnesium–aluminum hydroxide suspension to see an effect on these studies. This anti-acid suspension was used to neutralize the pH of the GI tract, thereby preventing digestion of the Cry protein (See Impact of Protein Digestibility) and eliminating a formidable element of the GI tract barrier. Additionally aluminum hydroxide is a known antigenic adjuvant (Mannhalter et al., 1985; Rimaniol et al., 2004) and therefore could be responsible for the effect observed when the Cry proteins were delivered. Another detail which calls into question the relevance of these results is that the mice were often dosed with 100 μg Cry1Ac protein/25 g mouse (∼4 mg/kg BW). While this may not seem like an exceedingly large dose at first glance, this amount of Cry1Ac translates into at least 50,000-fold greater exposure than estimates of Cry1Ab human intake (Hammond and Jez, 2011), which conservatively presumed that *Bt* maize grain was consumed uncooked. These experimental conditions clearly exceed any reasonable potential human dietary intakes. Furthermore, since maize grain is normally processed (e.g., by cooking) before human consumption, which denatures Cry proteins, the level of functionally active Cry protein in food products derived from processed maize has been estimated to be reduced by approximately two orders of magnitude (Section Impact of Heat on Stability). Thus the differences in dietary exposure between the aforementioned mouse study and humans would increase from 50,000 to 5,000,000 (Hammond and Jez, 2011).

In addition to the lack of relevancy to potential health risks from human consumption of Cry protein containing crops, an attempt to reproduce these studies (Vázquez-Padrón et al., 1999a,b, 2000a,b; Moreno-Fierros et al., 2000) in mice with Cry1Ab failed to detect anti-Cry IgG antibodies. Adel-Patient et al. (Adel-Patient et al., 2011) reported that IG administration of purified Cry1Ab protein had no impact on immune response in mice and confirmed the earlier reports of the immunogenicity of Cry1Ab administered by IP injection to mice, without any evidence of allergenicity. One potential explanation for this discrepancy in results is that Cry protein preparations used in the studies where antibody production was observed were contaminated with *E. coli* endotoxin. The Cry proteins used in these studies were produced and purified from *E. coli* but were not apparently checked for endotoxin contamination. Additionally, the routes of exposure and amounts of protein required to elicit a response were very different from the dietary exposure of Cry proteins in GM crops (See Human Dietary Exposure Assessment for Cry Proteins in *Bt* Crops). Given the low dietary exposures to Cry proteins from consumption of foods derived from *Bt* crops, the potential to induce or enhance an immune response in humans is unlikely (Guimaraes et al., 2010; Hammond and Koch, 2012).

### Cry Protein Binding to Mammalian Intestinal Cells

In contrast to the information presented in Section “Receptor-Mediated Selectivity of *Bt* Cry Proteins” on receptor-mediated selectivity, Vázquez-Padrón et al. (2000b) reported binding of Cry1Ac protein to BBMVs isolated from mouse small intestine. Unlike the specific toxin/receptor binding that occurs in target species, the binding appeared to be non-specific because of the non-physiologically relevant concentrations of Cry1Ac protein were used (1 μg Cry protein/1 μg BBMV). This concentration is many orders of magnitude higher than the potential dietary exposures a mammalian digestive tract might encounter from consumption of food derived from *Bt* maize (Hammond and Cockburn, 2008; See Human Dietary Exposure Assessment for Cry Proteins in *Bt* Crops) and may have been directly responsible for the observed binding simply due to the law of mass action (i.e., high levels of Cry protein drove non-specific binding of the protein to the mammalian BBMVs). The Cry1Ac concentration used in these studies was too high to be toxicologically relevant as it greatly exceeded levels toxic to target insect pests, where binding to receptors occurs at much lower concentrations (0.00001–0.001 μg Cry protein/μg BBMV). While one group reported that an insecticidal Cry protein bound to rat BBMV, they also note it did so with low affinity. Additionally, the bound protein could not be displaced by a 10,000-fold excess of unlabeled Cry protein (Hofmann et al., 1988a). Taken together, the low affinity and inability to displace the labeled protein with unlabeled protein indicates non-specific, or non-receptor-mediated, binding. This directly contradicts the claims of Vázquez-Padrón et al. (2000b) Similarly, other studies with BBMVs prepared from bovine intestines (Shimada et al., 2006) indicate Cry1Ab protein binds at low levels to the cytoskeletal protein actin, which is a structural protein, but not to extracellular proteins that have been identified as receptors for Cry protein binding on target insect mid-GI-tract epithelia. The ability of Cry proteins to bind actin is particularly relevant when considering the results of Vázquez-Padrón et al. (2000b) because BBMVs may form with intracellular proteins outside the vesicle (Haase et al., 1978), and actin is an intracellular protein. This technical limitation of the BBMV model makes it possible that the binding detected by Vázquez-Padrón et al. (2000b) was an artifact of the presence of actin rather than evidence for specific Cry protein receptors in the gut of mammals.

### Hematotoxicity

In another study, *Bt* microbial spore preparations containing various Cry proteins were reported to cause hematotoxicity in mice when administered by oral gavage (Mezzomo et al., 2013). It should be noted that this is in contrast to the absence of findings on many other animal studies, which contained comprehensive hematology data, following repeated dietary exposures to *Bt* microbials, individual Cry proteins, or *Bt* crops (see **Tables 3 and 4**). This result could be due to spore components other than Cry proteins as the cause of the hematologic effects because they used distilled water as the negative control and not an appropriate control (*Bt* spores lacking the Cry genes). Because the *Bt* spore formulation also contained many other bacterial proteins not present in the negative control (water), the cause of this observed effect is confounded with other components and the results cannot be specifically attributed to Cry proteins.

A few statistical re-analyses of data from 90-day toxicology studies with *Bt* crops have been published in which the authors alleged detection of new adverse effects that were missed in the original analysis (Séralini et al., 2007, 2009; Spiroux de Vendômois et al., 2009). Among the claimed findings in these statistical re-analyses were effects on the hematopoietic system. As noted by Bartholomaeus et al. (2013), the conclusions of these re-analyses and other studies (Velimirov et al., 2008; Séralini et al., 2012), have been comprehensively rebutted by both academic scientists and regulatory authorities world-wide (FSANZ, 2011b; Arjó et al., 2013; Grunewald and Bury, 2013). Moreover, the results of some of these studies (Velimirov et al., 2008) were actually supportive of an absence of adverse effects despite the conclusions of the authors (FSANZ, 2011b).

### Systemic Exposure in Susceptible Populations

A recent paper reported the detection of Cry1Ab protein in the serum of non-pregnant women, pregnant women, and the cord blood of their fetuses (Aris and Leblanc, 2011). Detection was performed with a commercially available ELISA immunoassay kit that has been validated for use in detecting Cry1Ab protein in grain/seed samples but not human serum. The authors did not report that they had validated the assay for Cry1Ab protein levels in human serum. The majority of reported serum “detects” were at or below the limit of detection (LOD) for the commercial kit used for grain detection. These points raise serious questions about the accuracy of the data, especially in light of previous reports that (1) a validated immunoassay to quantify Cry1Ab protein in plasma (LOD 1 ng/ml) was unable to detect Cry1Ab protein in any of the plasma samples collected from cows fed MON 810 maize at 70% w/w (dry matter) in the diet for 1 or 2 months (Paul et al., 2008), and (2) Cry1Ab protein could not be detected in the blood of pigs, which like humans are monogastric animals, after consuming diets containing 38% *Bt* maize for 110 consecutive days (Walsh et al., 2012b). Given that diet data were not reported for the test subjects, and that Cry1Ab is commonly used in both conventional and organic agriculture, linking their findings to GM crop consumption is speculative. A similar response to Aris and Leblanc (2011) has been rendered by risk assessment experts in Australia and New Zealand (FSANZ, 2011a).

## Environmental Testing and Assessment

Tiered testing with representative species to assess potential toxicity to NTOs was originally developed as a relevant and reliable process to make testing more efficient, avoid unnecessary tests for the assessment of conventional pesticides to NTOs, and enable mutual acceptance of data by regulatory authorities globally. Tiered testing is designed to first represent worst-case exposure scenarios in laboratory assays and only progress to more realistic scenarios if the lower-Tiered tests fail to indicate acceptable risk (Romeis et al., 2008). The Tiered hazard assessment approach for *Bt* crops was first developed by the American Institute of Biological Sciences in 1996 and confirmed as an acceptable method of environmental hazard assessment by a Federal Insecticide, Fungicide, and Rodenticide Act (FIFRA) Scientific Advisory Panel (SAP) on biopesticides in 1999. The SAP agreed that the Tiered testing and assessment approach was suitable for use with plant-incorporated protectants such as *Bt*, and testing should include beneficial invertebrates closely related to target species and/or likely to be present in GM crop fields and to meet existing regulatory requirements as well as protection goals (discussed further below). This has resulted in the development of a harmonized Tier I NTO battery that has been used in support of *Bt* crop registrations for approximately two decades (Carstens et al., 2014).

Species tested in the Tier 1 battery are selected to protect valued ecosystem services (e.g., pollination, decomposition, predation, and parasitism for biological control) or have a conservation interest. Surrogate species that are selected for testing typically meet three criteria, ecological relevance, reliability for laboratory testing with established and/or validated test guidelines, and potential sensitivity based on taxonomic relatedness to the target species, which has been a good predictor for biological activity for currently registered Cry proteins. Romeis et al. (2012) provided guidance on how to best use information that influences the scope of surrogate species testing for an insecticidal trait. When the mode of action is well characterized, the spectrum of activity is narrow and exposure is limited and/or the level and duration of exposure is well defined, the scope of NTO can potentially be reduced compared to the scenario when little is known about the mode of action, activity is broad spectrum and there are multiple routes of exposure at relatively high concentrations. Following this approach could limit the collection of hazard data to those species that are relevant for the ecological risk assessment.

Tier I high-dose studies can be broad in scope and allow control over experimental variables and exposure conditions that produce statistically reliable results by testing a sufficiently large number of organisms at a limit dose (Romeis et al., 2011). A limit dose is a single treatment level that is a sufficiently high exposure level (i.e., 10× a Tier I exposure estimate) that a large margin of safety is provided and a substance can be classified as non-toxic at realistic field exposure levels (Romeis et al., 2008). Importantly, lack of adverse effects in limit dose testing provides sufficient confidence to address uncertainties, allowing risk assessors to conclude that there is no unacceptable risk to the environment and that no further data are required (Rose, 2007). When screening studies conducted in a laboratory setting have suggested potentially unacceptable risk, additional higher-Tier studies can be designed to assess risk under more realistic exposure conditions (Romeis et al., 2008). For example, Tiers II–IV also provide definitive assessments by using laboratory studies with refined exposure estimates (Tier II), employing greenhouse or semi-field testing (Tier III), or utilizing field testing (Tier IV). These higher-Tier studies would only be sequentially implemented if unacceptable adverse effects were observed at the Tier I screening level.

In addition to invertebrate testing, mammalian and avian studies are performed. Mammalian studies used to support the food and feed safety assessment are also used to support the wild mammal assessment. Forty-two-day broiler growth studies are performed to assess chronic risk and short-term quail feeding studies are performed to support the avian assessment (EPA, 2010a).

After publication of Rosi-Marshall et al. (2007) which reported impacts of Cry1Ab on Caddis flies, EPA (2010a) started to require chronic aquatic invertebrate testing to assess potential risk to of aquatic invertebrates from exposure to residues from *Bt* crops. However, since the publication of Rosi-Marshall et al. (2007), numerous researchers identified issues with the relevance and reliability of the original caddis fly study (Swan et al., 2009; Jensen et al., 2010, summarized by Beachy et al., 2008; Parrott, 2008; Wolt and Peterson, 2010). Criticisms of the study included: (1) adverse effects were not caused by toxicity of Cry1A but, rather, by other differences between plant test substances (Jensen et al., 2010); (2) the abundance of *Trichoptera* in streams containing residues of Cry1A was not reduced (Chambers et al., 2007); and (3) while postharvest crop residue was identified as the most likely route of exposure (Carstens et al., 2012), aquatic exposure to biotech crops has been shown to be limited temporally and spatially with low to negligible exposure concentrations of Cry proteins in post-harvest crop tissues (Swan et al., 2009; Chambers et al., 2010; Jensen et al., 2010; Wolt and Peterson, 2010; Carstens et al., 2012). Consequently, EPA (2010d, 2014) stopped uniformly requiring additional aquatic invertebrate studies to assess hazard to aquatic invertebrate species.

To evaluate the predictive and protective capability of the Tier I NTO battery, Duan et al. (2010) performed a retrospective validation of the tiered approach for arthropod-active GM events. This meta-analysis concluded that Tier I laboratory studies have been accurate and conservative in evaluating the environmental safety of *Bt* crops. This same conclusion can also be reached by comparing laboratory and field results for Vip3Aa in corn (Dively, 2005; Raybould and Vlachos, 2011), Cry1Ac and Cry1F in soybean (EPA, 2014) and Cry1Ac and Cry2Ab2 in cotton (Whitehouse et al., 2005). These conclusions are also consistent with findings by Rauschen et al. (2010), who recommended that potential effects on key biocontrol agents (e.g., coccinellids) are most reliably assessed in lower-tiered lab studies because of the high level of natural variability observed in the field.

Over the past several years, a number of meta-analyses have been published examining the safety of *Bt* crops to NTOs. Marvier et al. (2007) performed an analysis across 42 field experiments and found that non-target invertebrates are more abundant in *Bt* cotton and *Bt* maize fields than in non-transgenic fields managed with chemical insecticides. Wolfenbarger et al. (2008) performed an analysis using 45 field studies across cotton, maize, and potato and found no uniform effects of *Bt* plant-incorporated protectants on the functional guilds of non-target arthropods. Duan et al. (2008) examined 25 studies that independently assessed potential effects of Cry proteins on honey bee survival and found that the Cry proteins did not negatively affect the survival of either honey bee adults or larvae in laboratory settings. Recently an update and review of the previous NTO meta-analyses was published by Naranjo (2009). This study focused on two environmental elements: effects on non-target invertebrates and changes in insecticide use patterns since the adoption of *Bt* maize and cotton. The review concluded that hazards identified in the laboratory are commonly an artifact of the experimental laboratory procedures and do not manifest in the field. For example, apparent negative effects can be caused by *Bt*-intoxicated prey or hosts because of their low quality, rather than as a direct result of *Bt* toxicity. Additionally, the analyses clearly demonstrated that potentially minor indirect negative effects of *Bt* crops demonstrated in the field are insignificant in comparison with alternative pest suppression measures based on use of traditional chemical insecticides. Taken together, this body of work demonstrates the high level of taxonomic specificity for the *Bt* proteins currently approved for cultivation.

In addition to the NTO assessment, the environmental assessment includes a comparative assessment between the *Bt* crop and the appropriate conventional control that is genetically similar but lacks the introduced trait to address unintended effects. This assessment is conducted under diverse geographies to capture the range of environmental conditions. Specific phenotypic, agronomic, and ecological characteristics are measured in the *Bt* crop and the conventional control to evaluate whether the introduction of the insect resistance has resulted in any changes that might cause ecological harm in terms of altered weed characteristics, susceptibility to pests, or adverse environmental impact. Additionally, environmental interaction data are collected in field experiments for *Bt* crop and the conventional comparator to evaluate potential adverse effects. These data include measurements of disease and insect susceptibility and crop damage under natural infestation pressure. Further to the agronomic and phenotypic evaluation, potential movement of transgenes from a GM crop plants into wild relatives is assessed for a new pest resistance gene in a new crop. This aspect of the assessment addresses uncertainty regarding the potential effect the *Bt* crop may have on plant populations in the wild. This concern has been considered for each of the *Bt* crops currently registered by the U.S. EPA (2010d). EPA determined in these assessments that there is no significant risk of gene capture and expression of any *Bt* endotoxin by wild or weedy relatives of soybean and corn in the U.S. territories. However, for cotton there is a possibility for gene transfer in locations where wild or feral cotton relatives exist. Therefore, EPA requires stringent sales and distribution restrictions on *Bt* cotton within these geographic areas to prevent outcrossing or hybridization from the crop to sexually compatible relatives.

## Assessing Potential Interactions between Introduced Proteins in Combined-Trait Crops to Support the Safety Assessment

To improve the efficacy, pest spectrum, and durability of crops producing plant incorporated protectants (PIPs), either products producing individual PIPs have been combined through conventional breeding or individual events have been produced that express more than one PIP. These products have been developed with PIPs that have been shown to act independently and do not exhibit cross-resistance (EPA, 2008, 2010b). The focus of this section on safety assessment for combined trait PIPs and the impact of combination of PIPs on target pests is outside the scope of this review and has recently been covered in a comprehensive review (Head and Greenplate, 2012).

As the number of transgenic traits has increased, the number of genes potentially introduced into a single GM crop product has also increased and has led to questions whether such traits might interact. According to EFSA (2009d), “In the case of GM plants obtained through conventional breeding of parental GM lines (stacked events), possible interactions between the expressed proteins, new metabolites, and original plant constituents should be assessed. If the potential for adverse interactions is identified, feeding trials with the GM food/feed are required.” The subject of protein interactions in GM crops has recently been examined in detail by Steiner et al. (2013), who present a decision-making process for evaluating whether the potential for adverse effects is present in combined-trait products. For over a decade, combinations of different Cry proteins have been introduced into food and feed crops to expand the number of insect pests that can be controlled and to reduce the potential for the development of insect resistance to the Cry insect-control proteins (Bates et al., 2005). Thus, the means by which combined-trait products are evaluated are particularly relevant to the deployment of *Bt*-containing GM products.

Many regulatory authorities have developed specific data requirements for import and/or cultivation approval that enable bridging to, and transportability of, existing product safety packages for the single trait arthropod-active products. As an example, the U.S. EPA (2009a,b) codified their requirements to bridge existing data from a previously registered crop with an insect-control trait to a new combined-trait insect-control product. It is implicit with the approach, provided no interaction (synergy) is observed with the largest combination of insecticidal products (e.g., A × B × C), that no interaction will be evident in smaller sub-combinations of the largest combined-trait product (e.g., A × B, B × C, A × C). The first requirement of the approach is confirmation that the presence and structure of the inserted material has been conserved in the combined-trait product. The second requirement is that expression of the arthropod-active trait is comparable in the single and combined-trait product, which confirms no biologically meaningful increase in exposure to NTOs. The third requirement is demonstrating a lack of synergism between arthropod-active traits using sensitive insect bioassays. Demonstrating the lack of synergism permits the application of the principle of independent assessment, which has a long history of use in toxicology. This principle states that if each substance in a mixture acts independently, and the substances are below their NOAELs, their toxicity can be assessed independently (EPA, 2009a,b). As applied to *Bt* crops, satisfying the requirements of this principle enables the use of existing safety studies performed separately for the individual products to assess the safety of the combined-trait product. EPA (2009a) has taken the position that for insecticidal traits with a record of proven safety, unless a greater than 10-fold degree of synergism is observed, there is no need to test for human health or non-target effects. However, in cases where *Bt* proteins with different, and especially novel, modes of action are being combined, the EPA (2009b) has stated that they would be justified in requiring additional testing for human health and NTOs effects for levels of synergism as low as fivefold.

Cry proteins that have been shown to be additive or demonstrate potentiation of toxicity in the target insect have not been associated with similar effects in non-target mammalian and avian species (Hammond et al., 2013). For example, Cry35Ab1 potentiates the activity of Cry34Ab1 against corn rootworm pests, but no evidence of toxicity was observed when the combination was tested in poultry or mice (McNaughton et al., 2007; Juberg et al., 2009), which would not be expected considering the margin of safety of each one individually. There has been no evidence of toxicity in animal feeding studies of individual Cry proteins or mixtures of Cry proteins in *Bt* microbial pesticide formulations (McClintock et al., 1995; WHO/IPCS, 1999; Betz et al., 2000; Siegel, 2001; Brake et al., 2003; Flachowsky et al., 2005; Taylor et al., 2005; McNaughton et al., 2007; OECD, 2007; Taylor et al., 2007; Federici and Siegel, 2008; Scheideler et al., 2008). As noted by Hammond et al. (2013), “These results are similar to what is observed in the safety assessment of mixtures of small molecules that have been individually assessed for safety: toxic (or pharmacological, metabolic, and pharmacokinetic) effects are not observed when the individual components of the mixture are administered at doses well below their toxicity thresholds (Seed et al., 1995; Groten et al., 1997).”

Likewise, there has been no evidence of any treatment-related adverse effects in 90-day rat toxicology studies carried out with *Bt* crops regardless of whether the crops contained one or more Cry proteins (**Table 4**). There was no evidence of synergistic toxicity in crops with combined (stacked traits), similar to what was observed years ago when *Bt* microbial formulations were tested in acute, subchronic, and chronic toxicity studies. These studies (**Table 4**) were undertaken early in the development of *Bt* microbial formulations when their acute mode of action was not clearly defined.

In a recent EFSA opinion on 59122 × 1507 × NK603, a combined-trait maize product expressing three Cry proteins and three herbicide-tolerance proteins, the panel stated, “As the composition of maize 59122 × 1507 × NK603 is comparable with that of non-GM maize varieties and the single events and also no indication for interaction between the newly expressed proteins was found, the GMO Panel is of the opinion that no additional animal safety studies are required” (EFSA, 2009c). As indicated by this and the other examples in **Table 5**, the weight of evidence for existing combined-trait products points to no apparent interactions among the traits. Even as new kinds of proteins (e.g., transcription factors) are introduced into crops, existing methods such as agronomic evaluation, compositional analysis, and hypothesis-based food and feed assessments can be used to test for protein–protein interactions in combined-trait products (Parrott et al., 2010; Steiner et al., 2013).

**Table 5:** **GM maize and cotton containing multiple Cry proteins positively evaluated by EFSA**.

Event(s)	Traits^a^	Reference
Maize		
MON810 × MON863	Cry1Ab, Cry3Bb1, NptII	EFSA (2005a)
MON810 × MON863 × NK603	Cry1Ab, Cry3Bb1, CP4 EPSPS, NptII	EFSA (2005b)
DAS-59122–7	Cry34Ab1/Cry35Ab1, PAT	EFSA (2007)
MON89034	Cry2Ab2, Cry1A.105	EFSA (2008)
MON89034 × MON88017	Cry1A.105, Cry2Ab2, Cry3Bb1, CP4 EPSPS	EFSA (2010c)
DAS-01507-1 × DAS-59122-7	Cry1F, PAT, Cry34Ab1/Cry35Ab1	EFSA (2009a)
MON810 × MON88017	CP4 EPSPS, Cry1Ab, Cry3Bb1	EFSA (2009b)
MON89034 × TC1507 × MON88017 × DAS-59122-7	Cry1A.105, Cry2Ab2, Cry1F, PAT, Cry3Bb1, CP4 EPSPS, Cry34Ab1/Cry35Ab1	EFSA (2010a)
*Bt* 11 × MIR604 × GA21	Cry1Ab, PAT, mCry3A, PMI, mEPSPS	EFSA (2010d)
Cotton		
DAS-24236-5 × DAS-21Ø23-5	Cry1Ac, Cry1F, PAT	EFSA (2010b)

*^a^EPSPS, 5-enolpyruvylshikimate-3-phosphate synthase; NptII, neomycin phosphotransferase II; PAT, phosphinothricin-N-acetyltransferase; PMI, phospho-mannose isomerase.*

## Benefits of *Bt* Crops

Adoption of *Bt* crops has been very rapid and attests to their popularity with farmers, who are the primary beneficiaries of their value. For the first time in 2012 and continuing in 2013, developing countries planted more hectares of GM crops than industrialized countries (James, 2013). Herbicide tolerance was the most widely used trait, but insect-resistant GM crops containing *Bt* proteins were planted on 76 million hectares in 2013, with most of that being maize and cotton. Almost 60% of *Bt* acreage was planted with stacked traits that include *Bt* with herbicide tolerance. It is noteworthy that *Bt* maize (MON810) is grown in five EU countries (Spain, Portugal, Romania, the Czech Republic, and Slovakia) and in 2013 four insect-resistant *Bt* brinjal (eggplant) varieties were approved for seed production and initial commercialization in Bangladesh. The start of limited cultivation of *Bt* brinjal is expected in 2014. Brinjal suffers considerable insect damage and as a result the adoption of *Bt* brinjal in Bangladesh is expected to improve the incomes of thousands of small-holder farmers and consumers in that country, as well as to reduce exposures to chemical insecticides and pesticide poisonings in the areas that adopt the technology.

The most widely used *Bt* vegetable crop is sweet corn. Shelton et al. (2013) compared sweet corn varieties grown in New York, Minnesota, Maryland, Ohio, and Georgia where the primary insect pest was *Helicoverpa zea*. They demonstrated that non-sprayed *Bt* varieties produced more clean, marketable ears than conventional isolines, even when the conventional corn was sprayed with chemical insecticides up to eight times. In the most comprehensive report that summarizes global data on GM crops from 1996 to 2012 (Brookes and Barfoot, 2014), the authors reported increased farm income, decreased pesticide use, and decreased greenhouse gasses (GHG), which have been attributed to use of GM crops. The greatest gain, as a result of reduced pesticide use, was for insect-resistant crops. There were 48 and 26% reductions in insecticidal active ingredients used on insect-resistant maize and cotton, respectively. These reductions in insecticide usage rates were for both developed and developing countries, but this benefit was most pronounced for *Bt* cotton grown in developing countries. Reduced GHG were primarily the result of the adoption of no-till practices. Although no-till is not necessarily associated with *Bt* crops, use of *Bt* crops is expected to lead to reduced fuel consumption due to fewer passes in the field for application of pesticides. As a result of decreased input costs and increases in crop yields, farm income was improved. Income in 2012 was increased by $6.7 billion and $5.5 billion for insect-resistant maize and cotton, respectively. For cotton, more than 75% of the increased income was in developing countries.

Adoption of *Bt* cotton has greatly reduced the abundance of targeted pests in cotton and in other crops with proximity to cotton that are impacted affected by polyphagous target pests (Naranjo, 2011). Additionally, reductions in insecticide use have facilitated and enabled integrated pest management (IPM) that, when appropriately managed can play an important part in suppression of other key and periodic pests in cotton. Furthermore, use of *Bt* cotton has helped to avoid or limit the use of broad-spectrum pesticides against the bollworm complex, which has reduced impacts to beneficial insects that are natural enemies to other cotton pests (Naranjo, 2011). Consequently, introduction of *Bt* cotton has enabled the success of biologically based IPM programs and for insecticide use to be nearly eliminated in a system previously dominated by chemical insect control measures.

### Reduced Chemical Insecticide Use and Applicator Exposure

A major advantage of *Bt* insect control has been a dramatic reduction in the need for application of conventional small-molecule, chemical insecticides over *Bt* commodity crops, most particularly corn and cotton. Adoption of *Bt* crops has resulted in a net reduction estimated at 50 million kilograms of predominantly organophosphate insecticides from 1996 to 2011 (Brookes and Barfoot, 2013); this is a considerable advantage in areas where small-holder agriculture is the norm and application is often by hand, as indicated by reductions in pesticide poisoning events (Hossain et al., 2004; Parrott et al., 2010; Kouser and Qaim, 2011). In Burkina Faso in West Africa, the planting of *Bt* cotton has enabled farmers to reduce the number of chemical insecticide sprays during a growing season from 6 to 2 applications (James, 2010). India reported pesticide use on *Bt* cotton has been cut at least in half. In a survey conducted for the years 2002–2008, Indian farmers reported *Bt* cotton use prevented at least 2.4 million cases of pesticide poisoning, saving $14 million (US dollar equivalent) in annual health costs (Kouser and Qaim, 2011). Further, there will be continued reductions in the large quantities of soil insecticides that were historically used for control of corn rootworms with the continued adoption and improved efficacy as coleopteran-resistant *Bt* maize adoption grows in the future.

### Reduced Mycotoxin Contamination of Grain

Another benefit of *Bt* insect control is the potential for reduced levels of mycotoxin contamination. In general, insect-damaged grain and other plant tissues are more susceptible to infection by fungi than intact tissues. Some of the fungi present in maize-growing areas and able to colonize maize include mycotoxin-producing fungi such as *Fusaria* (Hammond et al., 2004). *Fusarium* species produce the mycotoxin fumonisin, which is known to cause illness in farm animals and possibly in humans (Li et al., 2001; CAST, 2003; Marasas et al., 2004; Wu et al., 2004; Sun et al., 2007). Several studies conducted in diverse world areas, and under a wide range of environmental conditions (Hammond et al., 2004; Folcher et al., 2010; Ostry et al., 2010), have found that grain from *Bt* maize contains lower fumonisin levels than that from non-*Bt* maize. Reduced fumonisin contamination in *Bt* maize cannot be guaranteed, but a reduction in fumonisin contamination has generally been observed. This benefit is presumed to be a result of the decrease in insect damage, leading to fewer ports of entry for fungal infection (Hammond et al., 2004).

## Summary

Cry *Bt* proteins, whether in microbial pesticide products or expressed in *Bt* crops, have been used and consumed safely for decades. The levels of Cry *Bt* protein in GM crops are very low and are often reduced further by food processing. In addition, extensive testing of *Bt* proteins, single-*Bt* trait crops, and stacked trait crops containing *Bt* proteins has not revealed any harm to non-target insects and other non-target species, including humans. This environmental safety profile for *Bt* crops largely reflects the high level of taxonomic specificity that has been achieved with *Bt* crops currently approved for cultivation. Use of *Bt* crops provides benefits beyond insect control, such as significantly reducing small-molecule insecticide use for target pests controlled by *Bt* proteins, reducing applicator exposure to small-molecule insecticides, reducing greenhouse gasses emissions by minimizing field spraying with self-propelled sprayers or other motorized equipment, and by potentially reducing fumonisin levels in maize grain.

## Conflict of Interest Statement

All of the authors are, or were, employed by Monsanto Company. Monsanto Company produces and sells seeds some of which express *Bt* Cry proteins.
